# Paucity of Female Sexual Dysfunction Training in Urology and Obstetrics and Gynecology Residencies

**DOI:** 10.7759/cureus.106750

**Published:** 2026-04-09

**Authors:** Catherine Implicito, Sarah M Brink, Angelo Cadiente, Nafee Ullah, David Shin

**Affiliations:** 1 Department of Urology, Hackensack Meridian School of Medicine, Nutley, USA; 2 Department of Urology, Hackensack University Medical Center, Hackensack, USA

**Keywords:** curriculum, female sexual dysfunction, male sexual dysfunction, residency education, women's sexual health

## Abstract

Introduction

Despite its prevalence, female sexual dysfunction (FSD) is often overlooked in medicine, particularly when compared to male sexual dysfunction (MSD). Since both urologists and obstetricians and gynecologists manage FSD, their training curricula present a unique opportunity for comparison. This study aimed to evaluate the educational landscape by assessing the availability of FSD and MSD information on American Urological Association (AUA)- and American College of Obstetrics and Gynecology (ACOG)-accredited residency program websites, recognizing that website content may not fully represent actual curricular exposure.

Methods

The AUA and ACOG residency program lists were used to identify Urology (n=148) and Obstetrics and Gynecology (OBGYN) (n=228) residency program websites in 2024, respectively. Data was stratified by AUA section and ACOG district where appropriate. Chi-square and Fisher's Exact Test analyses were performed to compare content.

Results

Review of residency websites found that content on FSD was only present on 5.3% (n=12) of OBGYN and 1.4% (n=2) of Urology program sites, while MSD content was on 50% (n=74) of Urology sites. FSD and MSD content was readily accessible with a median of one click. Among programs with this content, 83% (10/12) of OBGYN residencies detailed FSD in their curriculum, compared to 45% (33/74) of Urology programs for MSD, and none (0/2) for FSD. The presence of a faculty member specializing in FSD was a significant predictor for the inclusion of FSD content on OBGYN residency websites (OR 4.06, 95% CI 1.29-12.76; p = 0.010). In contrast, faculty subspecialty in Andrology or Sexual Medicine did not significantly predict MSD content availability on Urology sites (p > 0.05).

Conclusions

Female sexual dysfunction content is lacking on Urology and OBGYN residency program websites compared to MSD content. Both Urology and OBGYN specialties should make stronger efforts to incorporate FSD education in their curricula to better address women's sexual health needs.

## Introduction

Female sexual dysfunction (FSD) encompasses a broad range of disorders affecting women’s sexual health, including orgasm, arousal, sexual desire, and sexual pain. FSD is a prevalent issue, impacting between 38% to 63% of women [1]. Despite its frequency, FSD receives less clinical emphasis, educational focus, and structured training compared to male sexual dysfunction (MSD). Disparities exist not only in clinical practice but in medical training as well. The onus of adequately treating women’s sexual health falls upon residency programs to sufficiently train their resident physicians on FSD. In a survey of University of Toronto resident trainees in Obstetrics and Gynecology (OBGYN), Urology, Psychiatry, and Family Medicine, only 12% of respondents reported that their residency programs provided adequate teaching and exposure to FSD compared to 25% of respondents reporting the same for MSD. Despite this discrepancy, 75% of respondents believed that FSD will be something that they will encounter in their future practice [2].

Inadequacies in training have been acknowledged by program directors (PDs), with 73% of Urology and OBGYN program directors in the United States reporting interest in additional FSD training in 2011. In the questionnaire distributed in the same study, 79% of program directors believed FSD training needed more improvements. Major barriers to adequate training included a lack of expert faculty and insufficient time and resources. At the time of data collection, 69% of programs did not have a standard protocol for FSD, and 51% “rarely or never” had faculty with specialized training in FSD. While both specialties acknowledged the need for more FSD education in residency, there were differences in training between OBGYN and Urology resident physicians. OBGYN programs were significantly more likely to encourage routine screening for FSD (63% vs. 24% in Urology), and OBGYN resident physicians received more training in female anatomy and physiology compared to their Urology counterparts (69% vs. 29%)​ [3].

However, since 2011, significant strides have been made by Urology and OBGYN programs to enhance both FSD and MSD training [4-6]. This study aims to evaluate and compare the presence of FSD and MSD content in Urology and OBGYN residency programs by analyzing their publicly available websites. By identifying the extent of FSD education in residency curricula, we hope to provide a clearer picture of the educational landscape and advocate for the inclusion of more comprehensive training. The ultimate goal is to ensure that physicians in both specialties are well-equipped to manage FSD, thereby improving the quality of care for women with sexual health concerns. This is necessary to determine if residency programs are providing adequate education in the management of FSD across both specialties. In doing so, this study will help to assess whether residency programs in both Urology and OBGYN are offering sufficient education in the management of FSD, ultimately ensuring that physicians are better prepared to address the sexual health needs of women.

## Materials and methods

The American Urological Association (AUA)’s 2024 residency program list was used to identify 148 Urologic residency programs within the United States. The American College of Obstetricians and Gynecologists (ACOG)’s 2024 residency program list was used to identify 228 OBGYN residency programs. Residency information was obtained from residency websites. Three reviewers independently analyzed each website to collect program director gender, program director subspecialty, and sexual medicine subspecialists on faculty between March and September 2024. In an effort to quantify the accessibility of online health resources, the website's content was systematically reviewed to determine the navigational effort, measured in clicks, needed to access information on female sexual dysfunction (“sexual health/function/dysfunction,” specifically related to females, “vaginismus,” “pelvic pain”) and male sexual dysfunction (“erectile dysfunction,” “impotence,” “men’s health,” “sexual health/function/dysfunction,” “prosthetics”). Information on “female pelvic health,” “pelvic floor dysfunction,” and “incontinence” was not included as information regarding FSD. Each program’s online curriculum was then examined to determine the mention of these topics in formal education.

Study data were collected and managed using REDCap electronic data capture tools (Vanderbilt University, Nashville, USA) hosted at our institution [7]. REDCap (Research Electronic Data Capture) is a secure, web-based software platform designed to support data capture for research studies, providing 1) an intuitive interface for validated data capture; 2) audit trails for tracking data manipulation and export procedures; 3) automated export procedures for seamless data downloads to common statistical packages; and 4) procedures for data integration and interoperability with external sources. Institutional Review Board protocol was not needed due to all information gathered publicly on residency websites. Data were stratified by AUA section and ACOG district, location, and population. A chi-square analysis was performed to examine associations between the presence of sexual dysfunction content on residency websites and faculty characteristics; however, analyses were limited to available website-derived variables and did not adjust for program-level confounders such as size or academic affiliation. In cases where chi-square analyses were not suitable, Fisher's Exact Test was alternatively utilized to ascertain an association. Additionally, trends in sexual dysfunction content were analyzed across OBGYN and Urology programs.

## Results

Of the 228 OBGYN residencies, 12 (5.3%) mentioned FSD on their program websites. Content was found on these websites with a median of 1 click (range 0-3) from the homepage. Of the 148 Urology residencies, only two (1.4%) program websites included FSD content, with a median of 1 click (range 0-2). For MSD, 74 (50%) Urology websites include MSD content, accessible with a median of 1 click (range 0-3). It is worth noting that both of the Urology programs that mentioned FSD also mentioned MSD on their websites. When examining the posted resident curriculum, 10 (83%) of the 12 OBGYN residencies with FSD content had FSD listed in their curricula. Of the Urology programs that included MSD content, 33 of them (45%) directly mentioned MSD in their curricula. Neither of the two Urology programs with FSD content mentioned FSD as part of their curricula.

Faculty composition was analyzed as a potential predictor of sexual dysfunction education. Of the 147 Urology programs, 28 identified female sexual dysfunction specialists, and 129 identified male sexual dysfunction specialists on faculty. For OBGYN, 45 programs specified a faculty member specializing in sexual dysfunction. The presence of an Andrology (𝛘2,1 = 0.167, p = 0.683; OR = 1.154, 95% CI: 0.580, 2.296) or Sexual Medicine subspecialist (𝛘2,1 = 0.089, p = 0.765; OR = 0.873, 95% CI: 0.358, 2.129) on faculty did not predict MSD content (p > 0.05) for a Urology program. An OBGYN FSD faculty member predicted FSD content (𝛘2,1 = 6.564, p = 0.010; OR = 4.060, 95% CI: 1.292, 12.761). In Urology programs, the gender of the program director did not provide evidence of being associated with either MSD (𝛘2,1 = 0.008, p = 0.930; OR = 1.036, 95% CI: 0.473, 2.269) or FSD (p = 0.391) content inclusion on residency website. In OBGYN programs, PD gender was also not associated with the presence of FSD on the residency website (p = 1.000).

**Figure 1:**
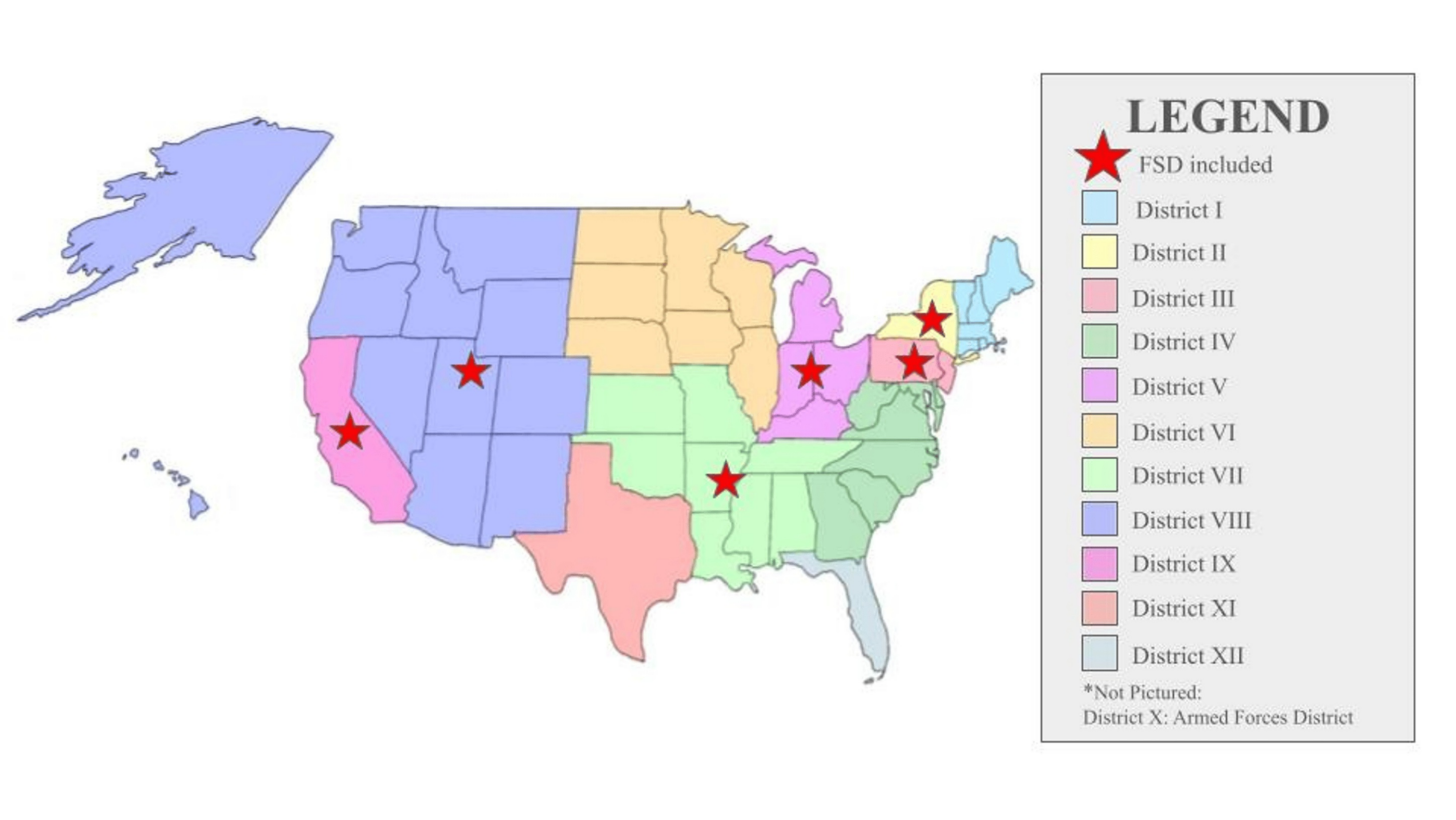
ACOG Districts with residency programs featuring FSD content on their websites (indicated by a red star) ACOG: American College of Obstetrics and Gynecology; FSD: female sexual dysfunction

**Figure 2:**
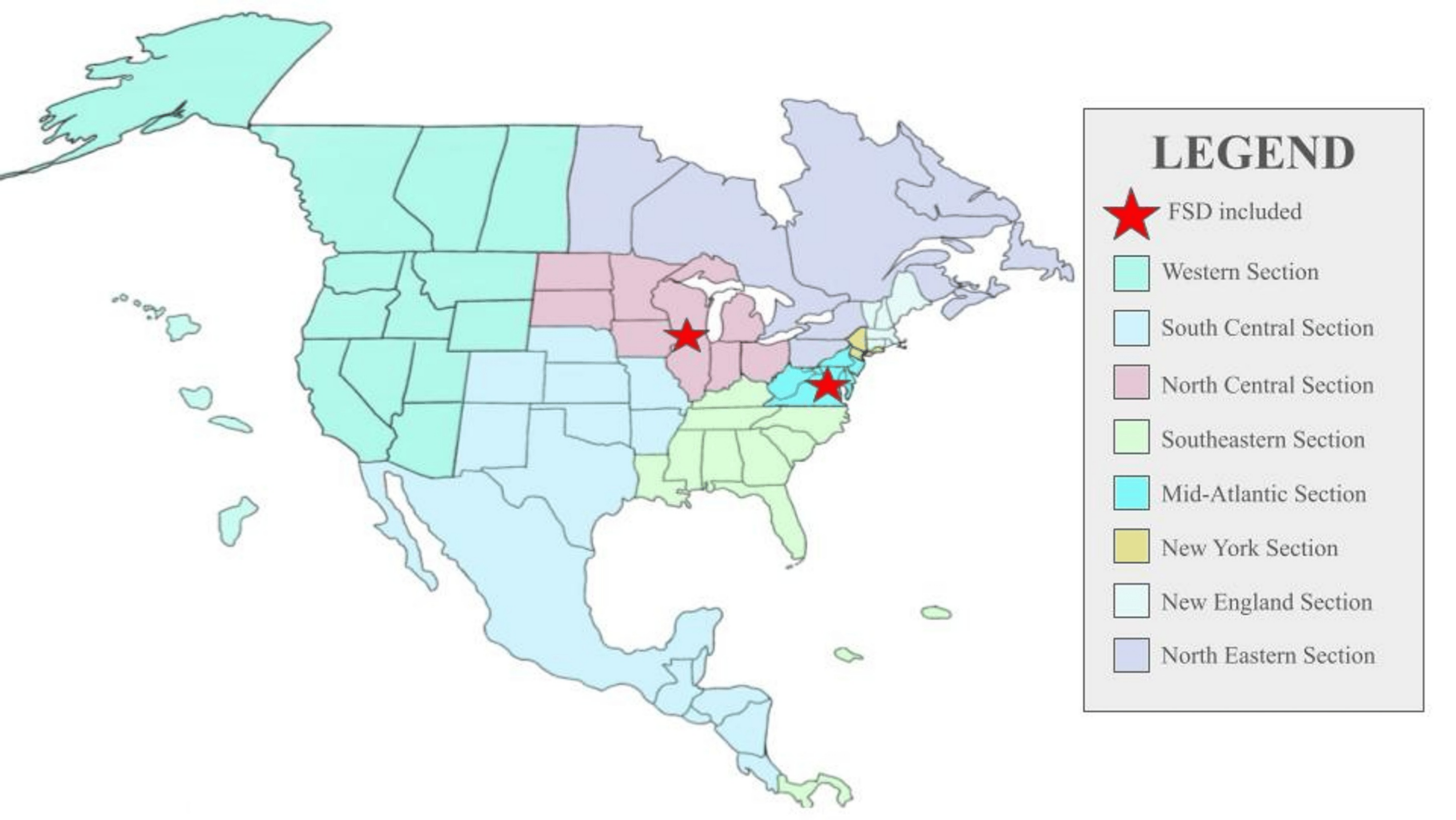
AUA Sections with residency programs featuring FSD content on their websites (indicated by a red star) AUA: American Urological Association; FSD: female sexual dysfunction

When comparing the geographic locations of programs displaying female sexual dysfunction content, OBGYN residency programs that included FSD spanned Districts II, III, V, VII, VIII, and IX (Figure 1). For the geographic location of Urology programs with FSD content, the two programs with FSD included on their website were from the Mid-Atlantic and North Central AUA sections (Figure 2). In contrast, MSD content was present across all AUA Sections.

## Discussion

There appears to be a significant gap in sexual dysfunction training in residency education. While half of Urology programs mention MSD, only 12% of OBGYN programs explicitly outline FSD content, indicating a clear deficit. Of the programs including content on their website, many do not report official inclusion into the curriculum. While both OBGYNs and urologists encounter patients who may suffer from FSD, the topic remains inadequately addressed in their training. Although MSD and FSD are included in the official AUA Core Curriculum, this emphasis does not appear to be consistently integrated into residency programs, based on information provided on residency program websites.

Female sexual dysfunction appears to be vastly forgotten in training for both specialties despite data suggesting that women are more likely than men to experience sexual dysfunction [8]. This may be because treatments for male sexual dysfunction are well-advertised and well-researched, but treatments for women are less apparent [9-11]. We suggest increased education and research on the topic to create well-trained physicians who can properly identify and manage female sexual dysfunction to help counteract this discrepancy. While some programs may reserve FSD education for informal clinical teaching, this can result in gaps in education amongst trainees.

The inclusion of FSD training in OBGYN programs is not equally distributed throughout the country. This geographic variability may reflect unmeasured sociocultural factors influencing how sexual health education is publicly presented, though this interpretation remains speculative and warrants further study [10]. In contrast, all sections of the AUA had MSD represented. While only two Urology programs report FSD content, 28 programs had female sexual function specialists on faculty, indicating that many programs have experts available to spearhead educational efforts in this area.

Another possible reason for scarcity within FSD education could be that both specialties assume that the other is providing sufficient coverage of the topic within their specialty education. Inter-disciplinary discussion could counteract this issue, as described in Millman et al. 2021, but ultimately, both specialties should be trained to treat this condition due to the overlap and prevalence in their patient population [2].

Limitations

One limitation of this study is its reliance on residency programs to have up-to-date, comprehensive, and accurate information. These programs may have informal education on these topics or may have only rudimentary or outdated information on their website. For instance, website deficiencies, such as non-functional hyperlinks or missing pages, do not definitively correlate with an omission in an educational curriculum. However, inadequate website maintenance can negatively influence a prospective applicant's perception of the program's quality and professionalism. Nevertheless, residency program websites are a resource that potential residency applicants use when deciding on programs to apply to or rank. It is important for potential residents to have an accurate understanding of the program's educational topics. Additionally, officially published information on residency websites and curricula provides a concrete commitment to educating on the topic that informal education does not provide.

It would be helpful to examine the personal accounts of resident trainees and programs on their perceived education on MSD and FSD via a survey. This should be looked at separately from official documents detailing the formal curriculum and information on residency websites, as an unofficial curriculum is not standardized. Additionally, this project did not delve into patients who do not fit the binaries of male and female. More research should be done to look at gender non-conforming patients.

## Conclusions

Despite the inclusion of FSD and MSD in the AUA Core Curriculum, individual Urology programs seem not to have adopted FSD education in the same manner as MSD. Similarly, FSD education in OBGYN programs remains insufficient, despite the specialty’s direct role in managing female sexual health. More concerted efforts are needed to incorporate female sexual function educational content in both urology and OBGYN programs to help train clinicians to address the sexual health concerns of women.
